# Identification of hub genes and pathways in hepatitis B virus‐associated hepatocellular carcinoma: A comprehensive in silico study

**DOI:** 10.1002/hsr2.2185

**Published:** 2024-06-17

**Authors:** Niloufar Sadat Kalaki, Mozhgan Ahmadzadeh, Atena Mansouri, Mohammadreza Saberiyan, Mohammad Hadi Karbalaie Niya

**Affiliations:** ^1^ Department of Cellular and Molecular Biology, Faculty of Biological Sciences Kharazmi University Tehran Iran; ^2^ Department of Biology, Science and Research Branch Islamic Azad University Tehran Iran; ^3^ Cellular and Molecular Research Center, Basic Health Sciences Institute Shahrekord University of Medical Sciences Shahrekord Iran; ^4^ Department of Medical Genetics, School of Medical Sciences Hormozgan University of Medical Sciences Bandar Abbas Iran; ^5^ Gastrointestinal and Liver Diseases Research Center Iran University of Medical Sciences Tehran Iran; ^6^ Department of Virology, School of Medicine Iran University of Medical Sciences Tehran Iran

**Keywords:** diagnostic biomarkers, HBV, hepatocellular carcinoma, PPI network

## Abstract

**Background and Aim:**

The hepatitis B virus (HBV) is one of the most common causes of liver cancer in the world. This study aims to provide a better understanding of the mechanisms involved in the development and progression of HBV‐associated hepatocellular carcinoma (HCC) by identifying hub genes and the pathways related to their functions.

**Methods:**

GSE83148 and GSE94660 were selected from the Gene Expression Omnibus (GEO) database, differentially expressed genes (DEGs) with an adjusted *p*‐value < 0.05 and a |logFC| ≥1 were identified. Common DEGs of two data sets were identified using the GEO2R tool. The Kyoto Encyclopedia of Genes and Genomes (KEGG) and gene ontology (GO) databases were used to identify pathways. Protein−protein interactions (PPIs) analysis was performed by using the Cytoscap and Gephi. A Gene Expression Profiling Interactive Analysis (GEPIA) analysis was carried out to confirm the target genes.

**Results:**

One hundred and ninety‐eight common DEGs and 49 hub genes have been identified through the use of GEO and PPI, respectively. The GO and KEGG pathways analysis showed DEGs were enriched in the G1/S transition of cell cycle mitotic, cell cycle, spindle, and extracellular matrix structural constituent. The expression of four genes (TOP2A, CDK1, CCNA2, and CCNB2) with high scores in module 1 were more in tumor samples and have been identified by GEPIA analysis.

**Conclusion:**

In this study, the hub genes and their related pathways involved in the development of HBV‐associated HCC were identified. These genes, as potential diagnostic biomarkers, may provide a potent opportunity to detect HBV‐associated HCC at the earliest stages, resulting in a more effective treatment.

## 1 INTRODUCTION

Hepatitis B virus (HBV) infection constitutes a prominent global health issue with a prevalence of about two billion. Despite the widespread availability of efficacious HBV vaccines, the persistence of approximately 350 million individuals as chronic HBV carriers worldwide underscores the enduring challenge posed by this infectious agent.^1^, ^2^ Moreover, infection with chronic HBV is known as the most common cause of hepatocellular carcinoma (HCC).^3^ HCC is the fifth most common cancer and the third cause of cancer‐related death in the world.^4^ In individuals afflicted by HBV infection and subsequent hepatic fibrosis, the potential emergence of HCC becomes a consequential clinical consideration.^2^ Several diagnostic methods, including serum alpha‐fetoprotein (AFP) levels and abdominal ultrasound tests, are utilized to detect early‐stage HCC. However, these methods frequently exhibit limited sensitivity in their ability to identify HCC during its initial phases, potentially due to the influence of environmental factors. Furthermore, liver biopsy, being an invasive procedure with inherent limitations in interpretation, frequently results in the diagnosis of HBV‐related HCC at advanced stages, consequently impeding the effectiveness of treatment.^5^

It has been predicted that by 2030, HCC will remain the third leading cause of cancer‐related death, and it is a disease with a poor prognosis.^6^ As a result, identification of the mechanisms responsible for the oncogenic activity of HBV could assist in the development of effective treatments for HBV‐related HCC. Understanding these mechanisms paves the way for further progress in the field of diagnosis and treatment in the future. Nowadays, the advancement of novel technologies, particularly in the realm of biomarker development, has significantly augmented the capacity for prognostication and prediction in HCC patient care. It is conceivable that these biomarkers can be used to detect HBV‐related HCC at very early stages without the need for imaging studies or a tumor biopsy.^2^

Therefore, utilizing molecular biomarkers with high sensitivity is considered an effective method for detecting HCC in its early stages, predicting disease progression, definitively diagnosing the disease, providing timely treatment, reducing treatment costs, and enhancing survivability. For example, Huang et al. indicated that the ACBD4 gene is significantly downregulated in HBV‐associated HCC patients and is correlated with a decrease in overall survival rate. They revealed that ACBD4 expression can serve as biomarkers of OS in HBV‐related HCC patients after hepatectomy.^7^ In addition, it is essential to use various bioinformatics methods to identify the primary genes and biological pathways that control tumor growth and advancement to uncover the molecular processes involved in cancer formation. This information may be used to create novel biomarkers or therapy approaches to enhance the prognosis of individuals with liver cancer. Gene expression analysis in cancer may independently predict survival and impact therapy decision.^8^ In this regard, data analysis was used to identify the key genes and pathways involved in the development of HBV‐related HCC in the present study. The hub genes that were highly expressed in HBV infection and HCC, the molecular pathways involved, and the target genes whose high expression reduced the survival rate were identified and discussed. Consequently, enhanced comprehension of the mechanisms implicated in the evolution and advancement of HBV‐related HCC empowers us to administer efficacious treatments. By promptly detecting the disease and intervening in a timely manner, prognosis and diagnosis through the utilization of these genes and their associated pathways have the potential to reduce mortality rates and optimize treatment strategies.

## 2 METHODS AND MATERIALS

### 2.1 Microarray data

Using GSE83148, which included gene expression data from 122 liver samples from chronic hepatitis B patients and six liver samples from healthy controls which was based on the GPL570 (Affymetrix Human Genome U133 Plus 2.0 Array) and GSE94660, an RNA‐seq data set which contains 21 HCC samples and 21 non‐neoplastic liver samples which was retrieved platform GPL16791 (Illumina HiSeq. 2500 [*Homo sapiens*]) in the Gene Expression Omnibus (GEO) (http://www.ncbi.nlm.nih.gov/geo/) were downloaded. GEO can be a useful database, since it provides hundreds of thousands of microarray gene expression data sets freely available for download and usage. The two data sets meet the following criteria: this experiment all met three criteria: (1) samples from human HBV‐infected liver tissue; (2) with case‐control group; and (3) sample number >40.

### 2.2 Common differentially expressed genes (DEGs) in HCC patients

An analysis of common DEGs was performed between HCC specimens and normal specimens was performed in GEO2R (https://www.ncbi.nlm.nih.gov/geo/geo2r/). The DEGs were screened according to adjusted *p* values < 0.05 and |log FC| (fold change) ≥1. Then, the common DEGs expressed jointly in the two data files were identified with the Venn diagram web tool (bioinformatics.psb.ugent.be/webtools/Venn/).

### 2.3 Functional enrichment analysis

Gene ontology (GO) (http://www.geneontology.org) is one of the most widely used methods in bioinformatics to annotate genes and their products on a large scale and is widely used in research. In this course, we will be covering three different aspects of biology, as well as biological process (BP), molecular function (MF), and cellular component (CC). By using the Kyoto Encyclopedia of Genes and Genomes (KEGG) (https://www.kegg.jp/), which is an extremely practical database resource, polymer experiments and genome sequencing can be done with ease. To determine which pathways a particular gene is enriched in based on the molecular information that is available, such as macromolecular data sets, one must use molecular information. To explore the functions of these DEGs, the DAVID database (https://david.ncifcrf.gov/) was used to perform GO term analysis at first. Then, we submitted 198 common DEGs into the Enrichr database to perform a KEGG pathway enrichment analysis. *p* < 0.05 was set to indicate a statistically significant difference.

### 2.4 Protein−protein interactions (PPI) network construction and performance analysis

For the purpose of determining the hub genes based on their PPI network, DEGs were imported to the STRING server (https://string-db.org; version 11.5). A PPI network with centrality parameters such as degree, betweenness, and closeness was used to determine the centrality parameters for the PPI network. To construct the PPI network, Cytoscape (version 3.6.0) was utilized as a tool, so the output file generated by STRING was imported into Cytoscape as a source for the analysis of significant genes in the network. As a result of using Cytoscape, hub genes were identified based on centrality parameters, including degree, betweenness, and closeness. Hub genes can be identified by using these genes as candidates, the output file was imported into the Gephi package to help screen and cluster the hub genes.

### 2.5 Verification and survival analysis

To enrich the characterization of these genes, we employed the “Enrichr” software‐a tool facilitating the analysis of modules derived from Gephi software. This utilization enables us to delve into the attributes of hub genes and explore their values effectively. Gene Expression Profiling Interactive Analysis (GEPIA), an interactive web server developed by Peking University, serves the purpose of analyzing and visualizing RNA sequencing expression data. To identify biomarkers associated with HCC, we conducted a differential mRNA expression analysis with the “single gene analysis” module of GEPIA.

### 2.6 Statistical analysis

We were able to calculate the values of the DEGs taken from the GEO data sets. Adjusted *p*‐values less than 0.05 were considered statistically significant and were counted as significant data. For GO and KEGG enrichment analyses, the threshold for significance is considered to be a *p*‐value < 0.05, which is considered the cut‐off point for significant results. In the Analysis‐Box Plots module of the GEPIA with the settings of *p*‐values < 0.05, log FC < 1, and a Match TCGA Normal to GTEx Data, allowing us to explore expression levels of genes associated with LIHC. Our biomarkers were selected with degree >45 and *p*‐value < 0.05.

## 3 RESULTS

### 3.1 Common DEGs in HCC patients

**Figure 1:**
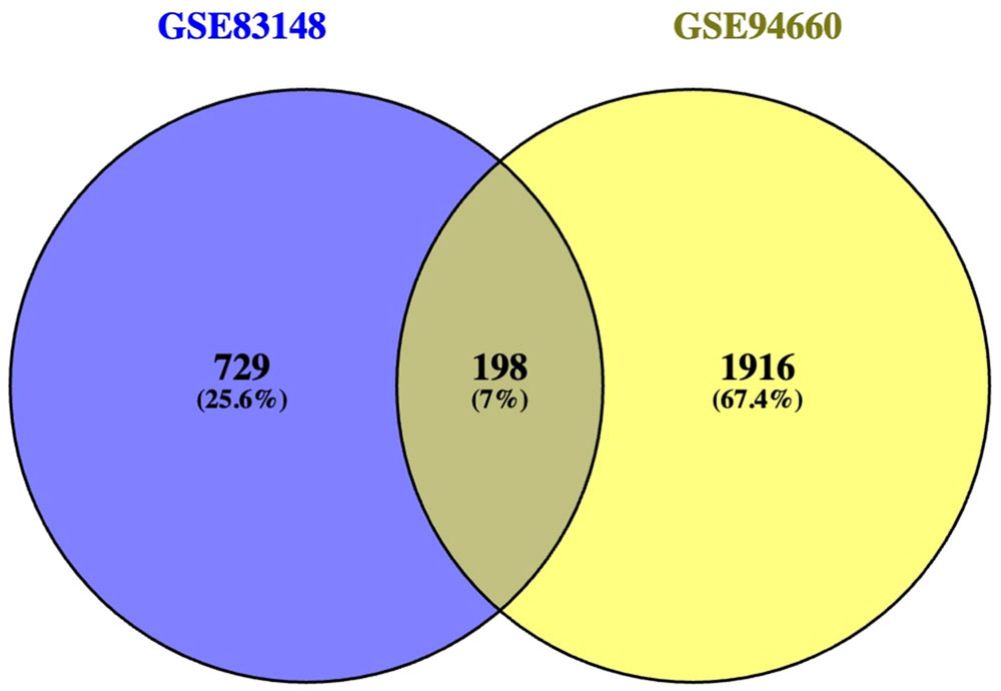
Identification of 198 common DEGs among GSE83148 and GSE94660 data sets by Venn diagram software. Different colors represent different data sets. DEGs, differentially expressed genes.

GSE83148 and GSE94660 were selected from the GEO database. Using the GEO2R online tool, we obtained 927 and 2114 DEGs from GSE83148 and GSE94660, respectively, adjusted *p* value < 0.05 and |log FC| ≥1 as the cutoff criteria to identify DEGs, then, we applied Venn diagram software to detect the common DEGs among the two data sets (Figure 1). Results showed that 198 common DEGs were obtained (a list of the 198 common DEGs is provided in Supporting Information S1: Table 1).

### 3.2 GO and KEGG pathway enrichment the common DEGs

GO annotation and KEGG pathways enrichment analysis were conducted through the DAVID database and Enrichr database, respectively. The top 10 enriched GO term and KEGG pathways are shown in Table 1. As shown in Table 1, GO BP analysis revealed that these 198 DEGs were significantly enriched in the G1/S transition of the mitotic cell cycle, mitotic sister chromatid segregation, chromosome segregation, and mitotic cell cycle phase transition, The top four significantly enriched CCs terms included spindle, kinetochore, cyclin‐dependent protein kinase holoenzyme complex, and mitotic spindle. For GO MF analysis, the top four significantly enriched terms were extracellular matrix (ECM) structural constituent, protein binding, chemokine activity, and platelet‐derived growth factor binding. Additionally, the top four markedly enriched pathways for these 198 DEGs were cell cycle, human T‐cell leukemia virus 1 infection, ECM‐receptor interaction, and viral protein interaction with cytokine and cytokine receptors.

**Table 1:** Functional and pathway enrichment analysis of the common DEGs.

Category	Term	Count	*p* Value
GOTERM_BP_DIRECT	GO:0000082~G1/S transition of mitotic cell cycle	8	3.56605035609663E−06
GOTERM_BP_DIRECT	GO:0000070~mitotic sister chromatid segregation	6	2.58200905140327E−05
GOTERM_BP_DIRECT	GO:0007059~chromosome segregation	8	3.41219711633171E−05
GOTERM_BP_DIRECT	GO:0044772~mitotic cell cycle phase transition	5	7.87113248359031E−05
GOTERM_BP_DIRECT	GO:0000079~regulation of cyclin‐dependent protein serine/threonine kinase activity	6	1.44251564141268E−04
GOTERM_BP_DIRECT	GO:0001503~ossification	7	1.61574155502457E−04
GOTERM_BP_DIRECT	GO:0006935~chemotaxis	8	1.70314932774602E−04
GOTERM_BP_DIRECT	GO:0031640~killing of cells of other organisms	7	2.19580406966191E−04
GOTERM_BP_DIRECT	GO:0007155~cell adhesion	16	2.75080469937276E−04
GOTERM_BP_DIRECT	GO:0007267~cell−cell signaling	10	3.53841839991809E−04
GOTERM_CC_DIRECT	GO:0005819~spindle	11	1.24320674807025E−06
GOTERM_CC_DIRECT	GO:0000776~kinetochore	10	1.97418504817842E−05
GOTERM_CC_DIRECT	GO:0000307~cyclin‐dependent protein kinase holoenzyme complex	6	5.67366371540544E−05
GOTERM_CC_DIRECT	GO:0072686~mitotic spindle	9	6.03463363997726E−05
GOTERM_CC_DIRECT	GO:0000775~chromosome, centromeric region	6	3.30619625206371E−04
GOTERM_CC_DIRECT	GO:0030496~midbody	9	3.7391894954945E−04
GOTERM_CC_DIRECT	GO:0005634~nucleus	77	0.0012684941777735500
GOTERM_CC_DIRECT	GO:0005604~basement membrane	6	0.0019456534558584200
GOTERM_CC_DIRECT	GO:0015630~microtubule cytoskeleton	8	0.002367388970954160
GOTERM_CC_DIRECT	GO:0005788~endoplasmic reticulum lumen	10	0.0024280317810708000
GOTERM_MF_DIRECT	GO:0005201~extracellular matrix structural constituent	11	0.0000009454919970515
GOTERM_MF_DIRECT	GO:0005515~protein binding	147	3.75883910263759E−05
GOTERM_MF_DIRECT	GO:0008009~chemokine activity	6	1.14979330928152E−04
GOTERM_MF_DIRECT	GO:0048407~platelet‐derived growth factor binding	4	1.35498266777386E−04
GOTERM_MF_DIRECT	GO:0048248~CXCR3 chemokine receptor binding	3	8.98486873086342E−04
GOTERM_MF_DIRECT	GO:1990837~sequence‐specific double‐stranded DNA binding	15	0.0010465795548514000
GOTERM_MF_DIRECT	GO:0019901~protein kinase binding	14	0.0016022040556511200
GOTERM_MF_DIRECT	GO:0008201~heparin binding	8	0.0017349747635131200
GOTERM_MF_DIRECT	GO:0000987~core promoter proximal region sequence‐specific DNA binding	5	0.0023238664167645900
GOTERM_MF_DIRECT	GO:0016538~cyclin‐dependent protein serine/threonine kinase regulator activity	4	0.003512810556673530
KEGG_PATHWAY	hsa04110:Cell cycle	13	0.000000178738363661
KEGG_PATHWAY	hsa05166:Human T‐cell leukemia virus 1 infection	11	1.98104521944018E−04
KEGG_PATHWAY	hsa04512:ECM‐receptor interaction	7	4.95605218722635E−04
KEGG_PATHWAY	hsa04061:Viral protein interaction with cytokine and cytokine receptor	6	0.005446431697476530
KEGG_PATHWAY	hsa05169:Epstein‐Barr virus infection	8	0.00789823594609545
KEGG_PATHWAY	hsa04115:p53 signaling pathway	5	0.00975613396479264
KEGG_PATHWAY	hsa05165:Human papillomavirus infection	10	0.012532645726405700
KEGG_PATHWAY	hsa04114:Oocyte meiosis	6	0.016379614160842600
KEGG_PATHWAY	hsa05215:Prostate cancer	5	0.024147672961561200
KEGG_PATHWAY	hsa04914:Progesterone‐mediated oocyte maturation	5	0.028386977938644300

Abbreviations: DEGs, differentially expressed genes; GO, gene ontology; KEGG, Kyoto encyclopedia of genes and genomes.

To learn more about the common DEGs found in HCC patients, GO and KEGG pathways associated with these DEGs were analyzed by Enrichr. It has been shown that several common DEGs play a significant role in the onset and progression of HCC, such as the cell cycle (*p*‐value: 4.219e−10), human T‐cell leukemia virus 1 infection (*p*‐value: 0.00001543), cellular response to chemokine (*p*‐value: 1.396e−7), mitotic cell cycle phase transition (*p*‐value: 1.967e−7), spindle (*p*‐value: 3.286e−8), collagen‐containing ECM (*p*‐value: 2.830e−7), chemokine activity (*p*‐value: 0.000006782), and cyclin‐dependent protein serine/threonine kinase regulator activity (*p*‐value: 0.000006782) (Supporting Information S1: Figure 1).

### 3.3 PPI network and Hub genes

**Figure 2:**
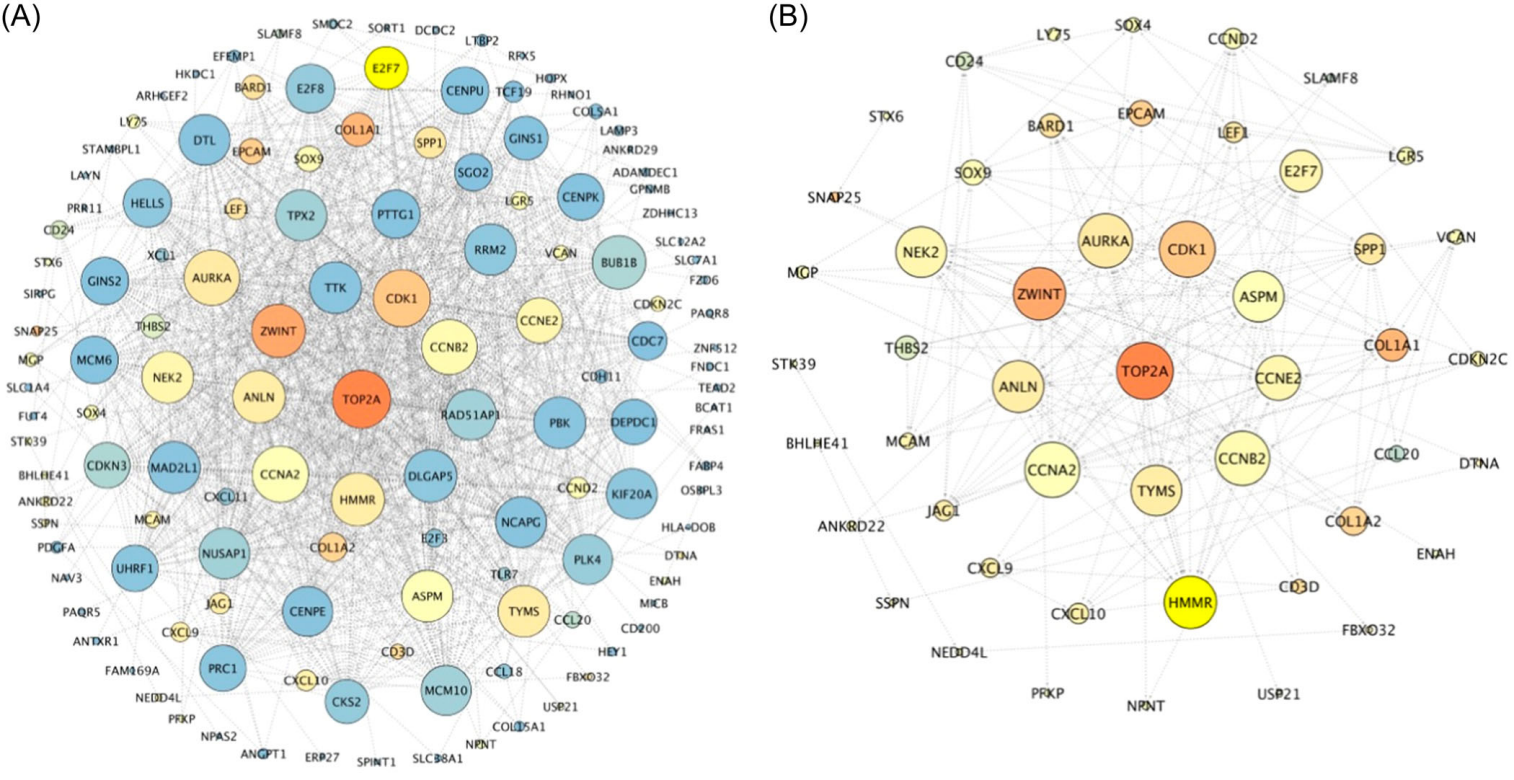
Protein–protein interaction (PPI) analysis. The size and the color of nodes represent the degree and betweenness, respectively. (A) PPI networks of the common differentially expressed genes (DEGs) from the GSE83148 and GSE94660, (B) PPI network of Hub genes of the common DEGs from the GSE83148 and GSE94660.

To construct a PPI network, we used the STRING database as a foundation (Figure 2A). To draw the PPI network, Cytoscape software was employed (Figure 2B). It is possible to identify strong molecular interactions that are influencing the progression of disease by analyzing PPI networks. A total of 134 nodes were identified as DEGs in the network (number of nodes: 134, clustering coefficient: 0.502, network centralization: 0.253). We ranked the hub genes according to their degree, closeness, and betweenness centrality (Supporting Information S1: Table 2). Using the STRING server and the parameters applied to identify the key hubs in the network, the common genes found in the top 49 genes were identified as being the network's key hubs (number of nodes: 49, clustering coefficient: 0.457, network centralization: 0.209) (Supporting Information S1: Table 3).

### 3.4 Clustering of Hub genes

**Figure 3:**
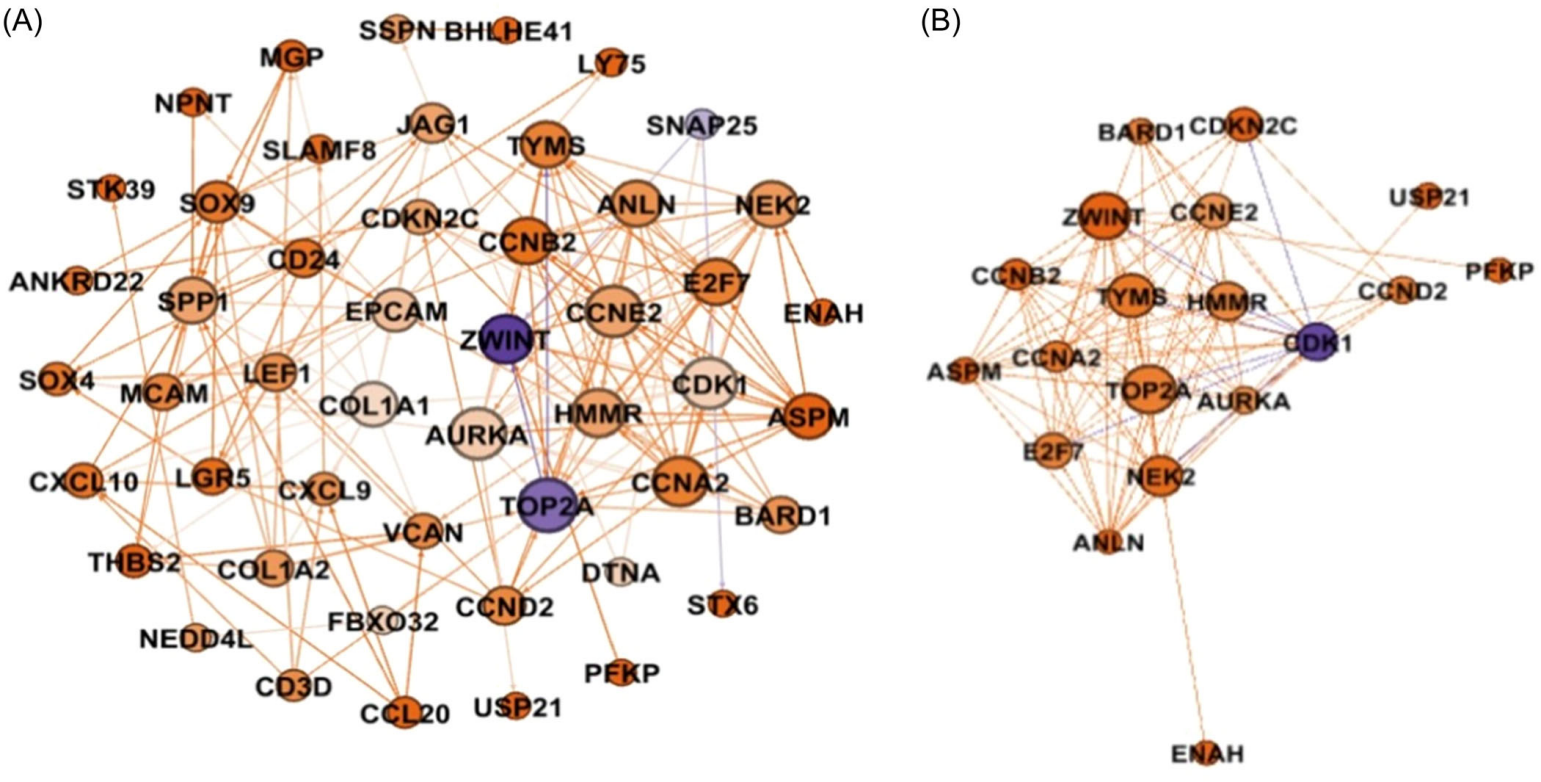
(A) Network visualization and analysis of hub genes from the PPI network of hub genes by Gephi. The size and the color of nodes represent the degree and betweenness, respectively. (B) Network visualization and analysis of hub genes in module 1. PPI, Protein−protein interactions.

Gephi 0.9.2 (www.gephi.com) was used to reconstruct the PPI network of the hub genes, and hub genes were clustered as a result of this process (Figure 3A).

Table 2 shows a graph of the interactions between hub genes. A total of five modules were identified as clusters within the network. The centrality parameters were then calculated for each module to determine its centrality. The interaction network of hub genes in module 1 had a strong interaction and high degree among each other (Figure 3B).

**Table 2:** The list of top ranked genes computed by Gephi.

Module 1		Module 2		Module 3		Module 4		Module 5	
Gene symbol	Degree	Gene symbol	Degree	Gene symbol	Degree	Gene symbol	Degree	Gene symbol	Degree
TOP2A	49	SNAP25	4	COL1A1	21	ANKRD22	4	FBXO32	2
CDK1	48	DTNA	2	SPP1	19	SLAMF8	3	NEDD4L	2
CCNA2	47	STX6	2	COL1A2	16				
CCNB2	46	SSPN	2	SOX9	14				
AURKA	46	BHLHE41	2	THBS2	13				
ZWINT	44			JAG1	11				
HMMR	44			LEF1	11				
ANLN	43			CXCL10	11				
TYMS	42			CXCL9	10				
NEK2	42			CD24	9				
ASPM	42			LGR5	9				
CCNE2	35			CCL20	8				
E2F7	32			EPCAM	8				
BARD1	13			MCAM	8				
CCND2	11			VCAN	7				
CDKN2C	7			SOX4	7				
USP21	2			CD3D	7				
ENAH	2			LY75	6				
PFKP	2			MGP	6				
				NPNT	3				

### 3.5 Verification of the hub genes

**Figure 4:**
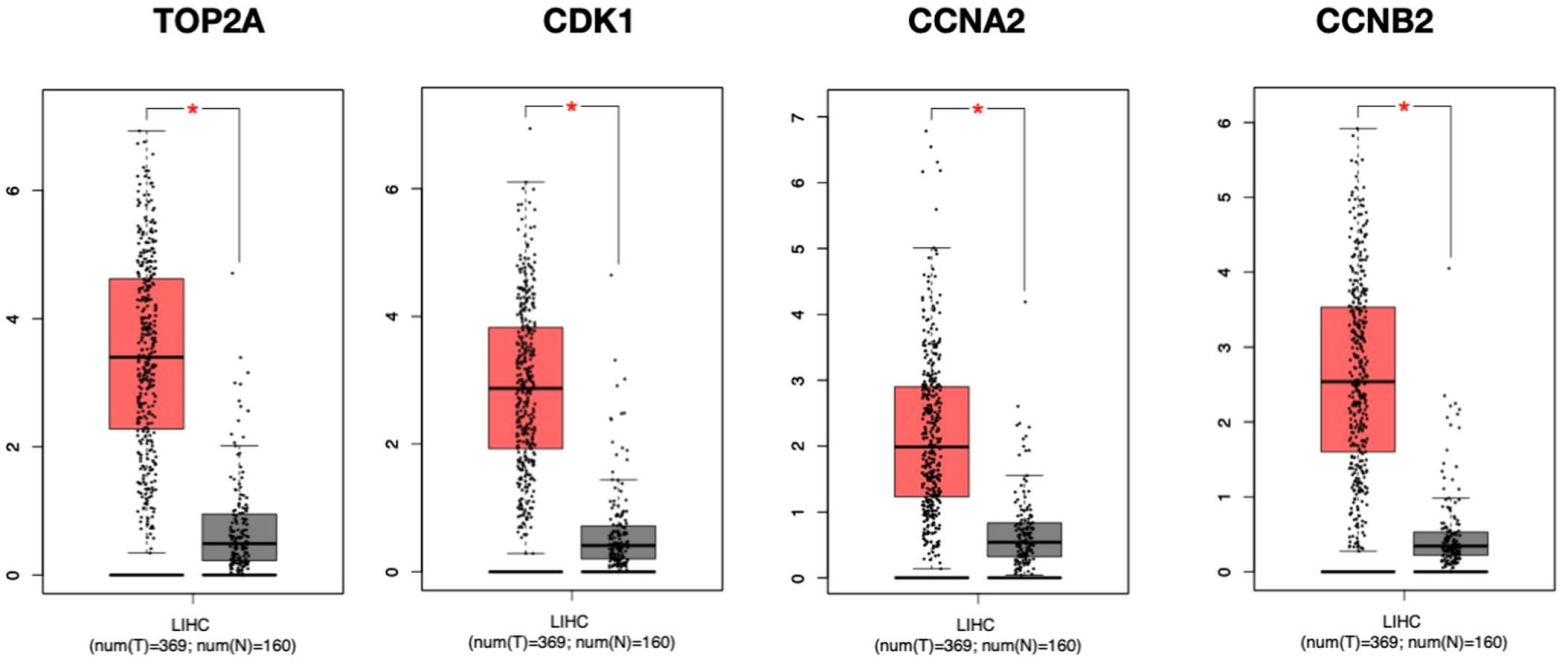
The Box plots of the core genes. TOP2A, CDK1, CCNA2, and CCNB2 showed a significant difference between normal and tumor samples. (*p < 0.05). CCNA2, cyclin A2; CCNB2, cyclin B2; CDK1, cyclin‐dependent kinase 1; TOP2A, topoisomerase 2‐alpha.

It has been shown by the GEPIA that some genes have significant prognostic value in HCC. The interaction network of hub genes in module 1 had a strong interaction and high degree among each other (Figure 3B). The expression of figure genes with high degrees in module 1 was higher in HCC samples than in normal samples such as DNA Topoisomerase 2‐Alpha (TOP2A), Cyclin‐dependent kinase 1 (CDK1), Cyclin A2 (CCNA2), and Cyclin B2 (CCNB2) in module 1 (Table 2). Therefore, these genes may be introduced as potential biomarkers for HCC, including TOP2A (*p* < 0.05), CDK1 (*p* < 0.05), CCNA2 (*p* < 0.05), and CCNB2 (*p* < 0.05) among other ones (Figure 4).

## 4 DISCUSSION

HCC is the predominant form of primary liver cancer in adults and is a leading cause of mortality in people with liver cirrhosis. HCC is strongly associated with HBV infection worldwide. Individuals with HBV are checked for HCC using AFP blood levels and abdominal ultrasonography every 6 months, yet HBV‐related HCC is still the primary cause of death in these individuals. Discovering novel biomarkers and regulatory mechanisms associated with HCC development in HBV‐infected individuals may improve the early detection, diagnosis, and treatment of HCC.^9^ In this regard, we identified 198 common DEGs between two gene expression data sets (GSE83148 and GSE94660) related to HBV‐associated HCC. GO and pathway analysis revealed these DEGs were significantly enriched in processes like G1/S transition of cell cycle mitotic, cell cycle, spindle, and ECM structural constituent. In addition, from the PPI network analysis, 49 hub genes were identified as potentially important regulators. Further analysis highlighted four specific hub genes—CCNA2, CCNB2, CDK1, and TOP2A—that showed significantly higher expression in HBV‐associated HCC tumor samples compared to normal samples in the GEPIA database. The results suggest CCNA2, CCNB2, CDK1, and TOP2A may serve as potential diagnostic biomarkers for early detection of HBV‐associated HCC.

Biomarkers may be found in tumor tissues, serum, and other body fluids. Several biomarker detection techniques using extremely specific recognition biomarkers have been created. ELISA, gel electrophoresis, SPR, Mass‐sensing BioCD protein array, SERS, colorimetric test, electrochemical assay, and fluorescence approaches are some biomarker detection techniques. Several approaches rely on traditional immunoassays where a capture antibody is attached to a solid support to capture the target, and a reporter antibody is used for assay read‐out. These methods are affected by the common issue of nontarget proteins adsorbing onto the surface of a biosensor.^10^ Most existing approaches have limitations like insufficient accuracy, sensitivity, and specificity needed for clinical diagnostic purposes. Moreover, blood‐based biomarkers show promise in cancer screening and might be used not only for assessing general population risk but also for evaluating treatment response and tracking recurrence.^11^ The primary objective of biomarker detection is to create a dependable, economical, and potent detection instrument for forecasting, identification, and tracking the reappearance of a certain illness. Several studies have used extensive bioinformatics analysis to discover central genes from PPI networks established using DEGs in several cancer types, including non‐small cell lung cancer (NSCLC),^12^ breast cancer,^13^ and colorectal cancer.^14^ It is worth noting that in the field of bioinformatics analysis, distinct research groups have the potential to uncover identical prognostic biomarkers utilizing disparate data sets.^13^, ^15^ This occurrence might potentially enhance the importance of data mining. The process of hepatocarcinogenesis triggered by HBV is intricate and influenced by several factors. In recent years, the use of bioinformatics techniques, such as microarray and next‐generation sequencing, has been prevalent in the analysis of large‐scale data. These approaches can be employed to investigate the underlying processes of HBV‐related HCC.

CCNA2, a cyclin family member, has a crucial function in the cell cycle and tumor development.^16^ In compatible with our findings, recent studies also showed that overexpression of CCNA2 is associated with progression of HCC linked to HBV infection.^17^, ^18^ CCNA2 interacts with CDK2 in the S phase and CDK1 at the G2/M transition to aid in DNA synthesis and chromosomal condensation. Research has shown that HBV often integrates into the CCND1 gene, which encodes a cyclin important in the cell cycle, and that CCNA2 is often elevated in HBV‐associated HCC.^19^ Dysregulation of CCNA2 may result in uncontrolled cell proliferation and promote tumor formation.^20^ As indicated in our results obtained through GO annotation and KEGG pathways enrichment analysis, cell cycle and G1/S transition of cell cycle mitotic are critical pathways, which were dysregulated through HCC progression affected by HBV infection. Indeed, the CCNA2 gene dysregulation is linked to the development of HCC in HBV‐infected individuals.^18^ HBV infection may trigger the activation of CCNA2 and other genes involved in the cell cycle, which enhances viral replication and contributes to the development of a premalignant phenotype.^21^ The upregulation of CCNA2 and other genes involved in the cell cycle is linked to the dysregulation of the Wnt/β‐catenin signaling system, which plays a crucial role in the growth, stem cell characteristics, and resistance to drugs in HCC.^21^ HBV infection may stimulate the production of host proviral factors such as HNF4A, PPARA, RXRA, and CEBPB, which enhance HBV gene transcription and replication.^22^ In addition, HBV's deregulation of TGF‐β expression seems to enhance the advancement of infected cells into the G2/M phase, linked to disrupted cell cycle control in HBV‐infected cells.^22^ Recurrent fusions of the CCNA2 gene and repeated rearrangements of the CCNE1 promoter region have been found in a specific subgroup of HCC characterized by the activation of CCNA2 or CCNE1 gene.^23^ Moreover, CCNA2 has been associated with cancer aggressiveness, recurrence, metastasis, and chemoresistance in many kinds of cancer. CCNA2's role in tumor formation suggests it might be used as a diagnostic indicator and treatment target for HCC and other types of cancer.^16^ The precise ways in which HBV disrupts CCNA2 and other cell cycle genes in HCC patients are not completely known. However, our results indicate that the disruption of CCNA2 and other cell cycle genes is crucial in the progression of HCC in HBV‐infected individuals.

For instance, CCNB2, another cell cycle‐related gene,^24^ was overexpressed according to our analysis data in HBV‐associated HCC patients in compare to healthy individuals. CCNB2, a cyclin B family member, is crucial for cell proliferation and tumor advancement, especially in breast cancer and HCC associated with chronic HBV infection.^24^ CCNB2 plays a role in regulating the transition from the G2 phase to the M phase by activating CDC2. Inhibiting CCNB2 results in the arrest of the cell cycle.^25^, ^26^ Research has shown that CCNB2 is often increased in HBV‐associated HCC and breast cancer, as well as in other human cancers such as lung and liver cancer, and its dysregulation may result in uncontrolled cell proliferation, promoting tumor formation.^26^ Suppressing CCNB2 has been shown to induce cell death and result in cell cycle arrest in the S phase, ultimately causing the malfunction of the G2/M checkpoints. Recent research has recognized CCNB2 as a promising noninvasive biomarker for breast cancer in peripheral blood mononuclear cells.^27^ Therefore, in regards to recent investigations and the current study, CCNB2's role in carcinogenesis suggests it might serve as a diagnostic marker and therapeutic target for treating HCC and other malignancies.

CDK1, which is necessary for cell division and growth, plays a vital role in regulating the cell cycle.^28^ As our findings released, CDK1 is a hub gene with increased expression levels in HBV‐associated HCC tumors. It is crucial in facilitating the transitions between G2/M, G1/S, and G1 phases. CDK1 phosphorylates many substrates related to the initiation and completion of mitosis, including BORA, MLL5, GWL, and CDCA5.^28^ As recent reports, CDK1 dysregulation is linked to carcinogenesis in many types of cancer, such as HCC associated with chronic HBV infection. Research has shown that CDK1 is excessively expressed in tumor cells, such as HCC.^29^

Interestingly, Zhu et al. found a new circular RNA named HBV_circ_1, generated by HBV, was discovered in HBV‐infected HepG2.2.15 cells and HCC tissue associated with HBV. The microarray study of 68 HCCT samples revealed a considerably increased abundance of HBV_circ_1 compared to paracancerous tissues.^30^ Furthermore, the survival rate of patients who tested positive for HBV_circ_1 was notably lower than that of patients who tested negative for HBV_circ_1. Transient expression of HBV_circ_1 promoted cell proliferation, motility, and invasion while suppressing apoptosis in vitro. Moreover, ectopic HBV_circ_1 expression led to an increase in tumor growth in live organisms. HBV_circ_1 was verified to interact with CDK1 to control cell proliferation. The findings indicate that the development of HCC might be facilitated by the interaction between HBV_circ_1 and CDK1. The results revealed a unique insight into the development and progression of HBV‐related HCC and identified a potential target for therapeutic medication development.^30^ However, the exact mechanism by which HBV affects CDK1 expression and leads to HCC progression is still not fully understood and requires further studies. Moreover, it has been suggested that CDK1 is a predictive biomarker in HBV‐related HCC, and its overexpression is associated with a negative prognosis and the advancement of tumors.^31^ Increased levels of CDK1 expression are linked to more advanced tumor grades and stages, suggesting its role in promoting cancer aggressiveness. Moreover, the expression of CDK1 has been associated with the infiltration of immune cells in tumors, emphasizing its importance as a prognostic indicator in HBV‐induced HCC.^32^

In addition, inhibiting or targeting CDK1 may improve cancer therapy by overcoming immune evasion mechanisms.^33^ Targeting CDK1 has been identified as a promising therapeutic approach for treating cancer. Several small compounds have been created to specifically block CDK1, thereby halting the proliferation of cancer cells. Blocking CDK1 may cause tumor cells to stop dividing and undergo a programmed cell death pathway, making it a potential target for cancer treatment.^34^ The significant presence of CDK1 in several types of cancer highlights its potential as a target for creating new cancer therapies that may be widely applied.^28^

Since CDK1 can regulate other cell cycle‐related genes such as TOP2A, it was expected that TOP2A was upregulated in HBV‐associated HCC patients in comparison to the healthy group.^35^ TOP2A, also known as DNA topoisomerase II alpha, is an essential enzyme involved in several biological activities such as DNA replication, transcription, and chromosomal segregation in mitosis.^36^ It is a distinctive indicator of cell growth and is involved in the development of cancerous tumors. TOP2A resolves topological restrictions during DNA replication and transcription by temporarily cutting both DNA strands to produce double‐strand breaks (DSBs).^37^ This action facilitates the unwinding and disentangling of the DNA strands, a process critical for the aforementioned cellular functions.^37^ Failure to repair these DSBs may lead to cytotoxic damage, genomic instability, and cell death. This can result in chromosomal translocations and mutations that promote the development of cancer.^38^ In addition, high levels of TOP2A have been linked to more aggressive tumor behavior and later disease stages in several types of cancer such as HCC,^38^ breast cancer,^36^ and NSCLC.^39^ Furthermore, upregulation of TOP2A is associated with a negative prognosis, increased tumor grades, and stages, suggesting its involvement in cancer aggressiveness.^39^, ^40^ Notably, chemotherapy that targets TOP2 enzymes, such as TOP2A, is often used to treat both solid tumors and blood cancers.^40^ However, the effectiveness of TOP2 inhibitors as primary chemotherapeutic agents is counteracted by the potential for secondary cancers and heart damage, underscoring the need for more accurate targeted approaches.^41^, ^42^ Research indicates that TOP2A might serve as a novel prognostic biomarker and therapeutic target for HCC. However, more studies and clinical trials are required to elucidate its regulatory processes.^43^

Currently, there has been little advancement in understanding the pathways that cause cancer in HBV infection. Notable events include enhanced TERT or TP53 mutation, activation of Wnt, mTOR/PI3K/Akt, and Ras/ERK signaling pathways, and exhaustion of CD8+T cells. There is increasing evidence that the abnormal composition of the matrisome plays a role in how the tumor microenvironment supports the development, advancement, and spread of HCC.^44^, ^45^ The matrisome consists of fundamental ECM components like as collagens, glycoproteins, and proteoglycans, as well as ECM‐related proteins such as ECM regulators, ECM‐affiliated proteins, and secreted factors.^46^ The matrisome of two mice models of HCC has distinct compositions, suggesting that ECM modification during HCC initiation is dependent on the individual cause.^47^ The matrisome of human intrahepatic cholangiocarcinoma has been identified, however, the matrisome of human HCC, particularly HBV‐related HCC, has not been discovered yet. As mentioned above, DEG genes (CCNA2, CCNB2, CDK1, and TOP2A) are increased in ECM structural constituents. Prior research has shown that the ECM of the liver undergoes remodeling during HBV infection. The hepatitis B e antigen stimulates hepatic stellate cells (HSCs), leading to abnormal ECM synthesis. Hepatitis B x protein (HBx) enhances the activity of matrix metalloproteinases and promotes the migration of HCC cells.^48^ Alterations in the extracellular milieu caused by HBV infection stimulate intracellular signaling pathways. The oncogene collagen triple helix repeat containing‐1 promotes the advancement of HBV‐related HCC by activating hypoxia‐inducible factor 1 alpha and vascular endothelial growth factor via the PI3K/Akt/mTOR pathway.^49^ Therefore, identification and characterization of the matrisome in relation to HBV‐related HCC are urgently required despite existing studies.

The present study has effectively identified key genes and signaling pathways that are essential in the development of HBV‐related HCC. The characterization presented in this study has significantly enhanced our understanding of the underlying processes involved in the development and recurrence of HBV‐related HCC. As a result, this research provides important insights into potential targets for early diagnostic interventions and timely treatment approaches for HBV‐related HCC. Nevertheless, the research does demonstrate some limitations. It is crucial to validate the validity of the discovered genes and signaling pathways by providing evidence of their practical functions in clinical samples. The present conclusions are based only on bioinformatics analysis, highlighting the need for empirical verification using clinical samples. Moreover, it is important to highlight that investigations of this kind need rigorous analytical examination and careful deliberation. The conducted statistical analysis has effectively identified a group of key hub genes that are designated for inclusion in databases. However, it is essential to recognize that the existing data set is incomplete, necessitating the need to update this information to assure the accuracy of future studies. Hence, while this analysis has produced noteworthy insights, its findings need verification via empirical clinical studies and meticulous data updates to foster a strong foundation of scientific knowledge.

In summary, this study utilized an integrative bioinformatics approach to identify critical genes and pathways associated with the progression of HBV‐related HCC. The dysregulation of cell cycle control and ECM remodeling appear to play major roles in promoting hepatocarcinogenesis triggered by chronic HBV infection. Four hub genes, CCNA2, CCNB2, CDK1, and TOP2A, were found to be consistently overexpressed in HBV‐positive HCC samples and may have utility as diagnostic biomarkers. Further experimental validation of these genes as biomarkers in clinical samples is warranted. A better understanding of the key mechanisms driving HBV‐HCC development will pave the way for biomarker‐based early detection and novel therapeutic approaches to improve patient outcomes.

## 5 CONCLUSION

In conclusion, this study identified 198 common DEGs that are dysregulated in HBV‐associated HCC using bioinformatics analysis. Further PPI network analysis highlighted 49 hub genes that may play critical regulatory roles. In particular, CCNA2, CCNB2, CDK1, and TOP2A were found to be significantly upregulated in HBV‐positive HCC tumor samples and may serve as promising diagnostic biomarkers. GO and KEGG pathway enrichment analysis also revealed that cell cycle regulation is profoundly disrupted, facilitating uncontrolled cell proliferation in HBV‐induced liver carcinogenesis. Taken together, these findings provide important insights into the key genes and pathways driving HBV‐related hepatocarcinogenesis. The hub genes identified, especially CCNA2, CCNB2, CDK1, and TOP2A, represent promising biomarker candidates and potential therapeutic targets worthy of further investigation for improving early HCC detection and treatment outcomes.

## AUTHOR CONTRIBUTIONS

**Niloufar Sadat Kalaki**: Visualization; validation; writing—original draft; investigation; software; resources. **Mozhgan Ahmadzadeh**: Conceptualization; investigation; methodology; writing—review and editing. **Mohammadreza Saberiyan**: Conceptualization; methodology; investigation; validation; writing—review and editing; data curation; supervision. **Mohammad Hadi Karbalaie Niya**: Data curation; validation; writing—review and editing; project administration; funding acquisition. All authors have read and approved the final version of the manuscript.

## CONFLICT OF INTEREST STATEMENT

The authors declare no conflict of interest.

## ETHICS STATEMENT

Ethics approved by the Ethical Committee of Iran University of Medical Sciences, Tehran, Iran by the code No: IR.IUMS.REC.1402.692.

## TRANSPARENCY STATEMENT

The lead author Mohammadreza Saberiyan, Mohammad Hadi Karbalaie Niya affirms that this manuscript is an honest, accurate, and transparent account of the study being reported; that no important aspects of the study have been omitted; and that any discrepancies from the study as planned (and, if relevant, registered) have been explained.

## Supporting information

Supporting information.

## Data Availability

The authors confirm that the data supporting the findings of this study are available within the article [Dr. Mohammadreza Saberiyan and Dr. Mohammad Hadi Karbalaie Niya] its supplementary materials. [Dr. Mohammadreza Saberiyan and Dr. Mohammad Hadi Karbalaie Niya] had full access to all of the data in this study and take complete responsibility for the integrity of the data and the accuracy of the data analysis.
