# Supplementary material for: Identification of hub genes and pathways in hepatitis B virus‐associated hepatocellular carcinoma: A comprehensive in silico study
Source: Health Sci Rep. 2024 Jun 17;7(6):e2185. doi: 10.1002/hsr2.2185 (PMC11183944; doi:10.1002/hsr2.2185)
Supplement: Supplementary file 1 — Supporting information. [file HSR2-7-e2185-s001.docx]

**SUPPLEMENTARY MATERIALS**

**Supplementary Figures**

| 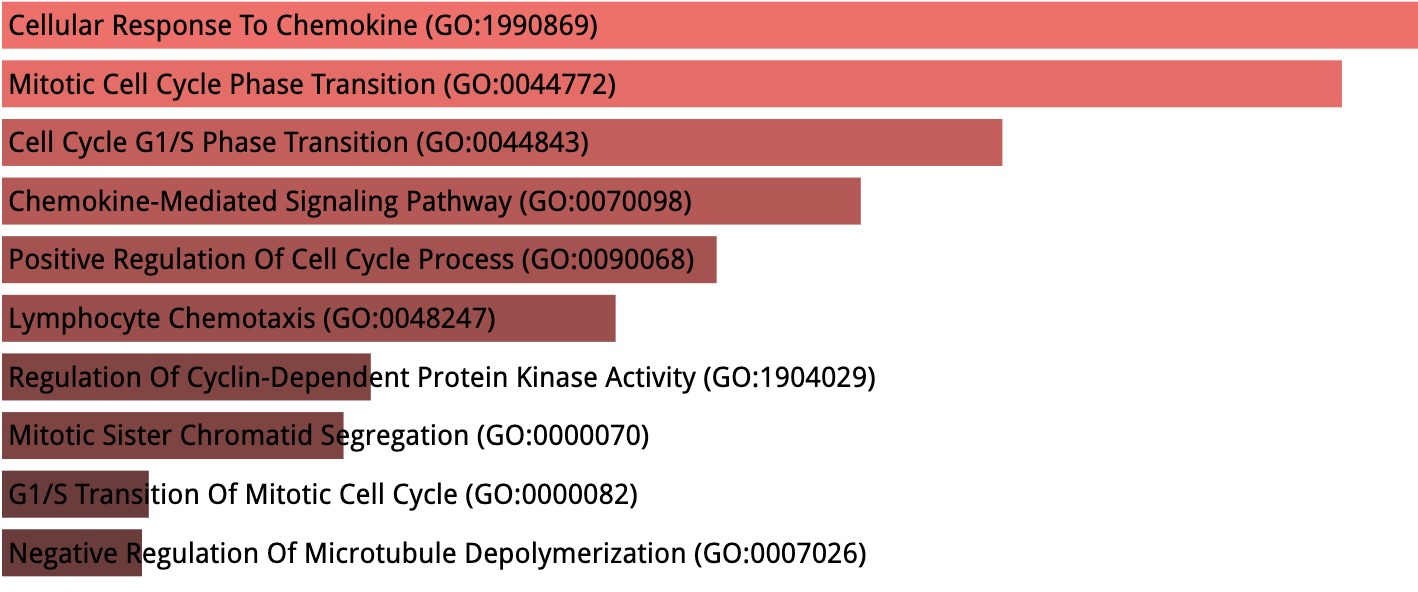 | 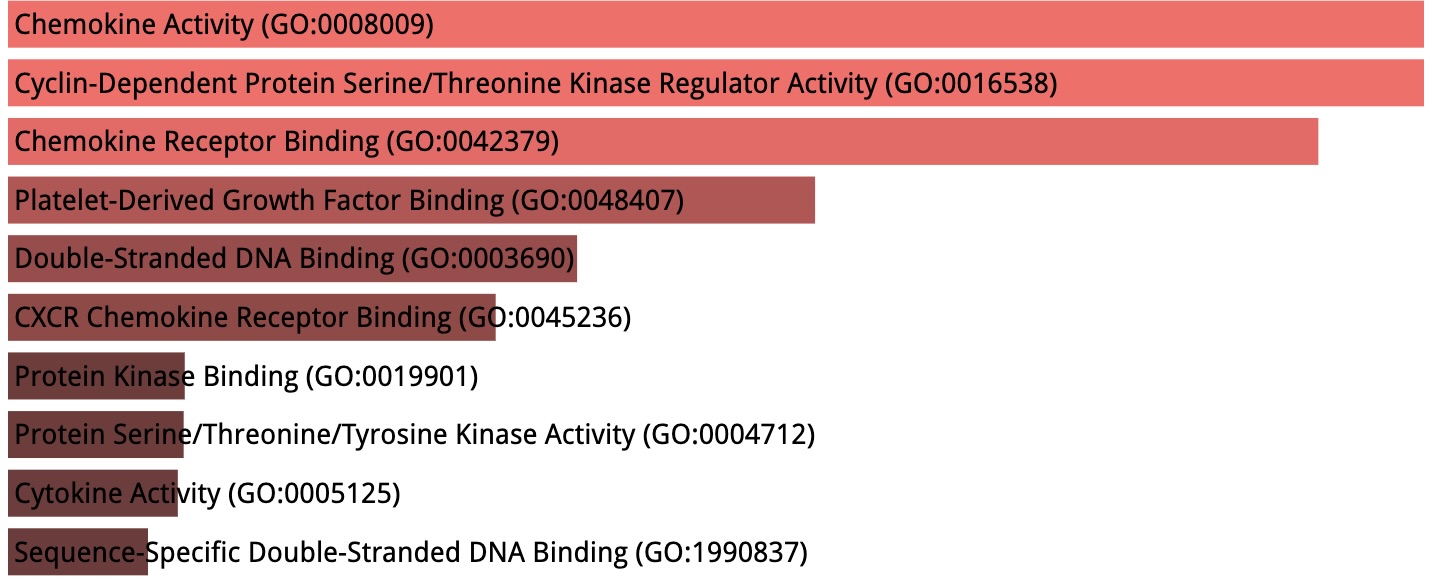 |
| --- | --- |
| 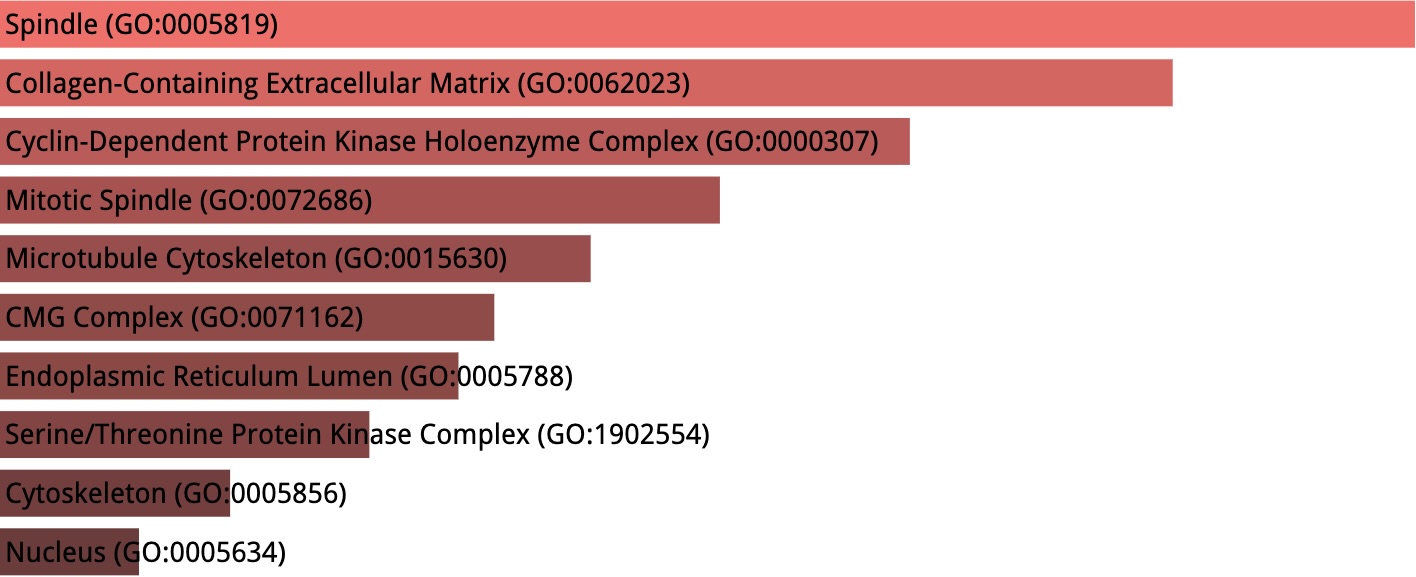 | 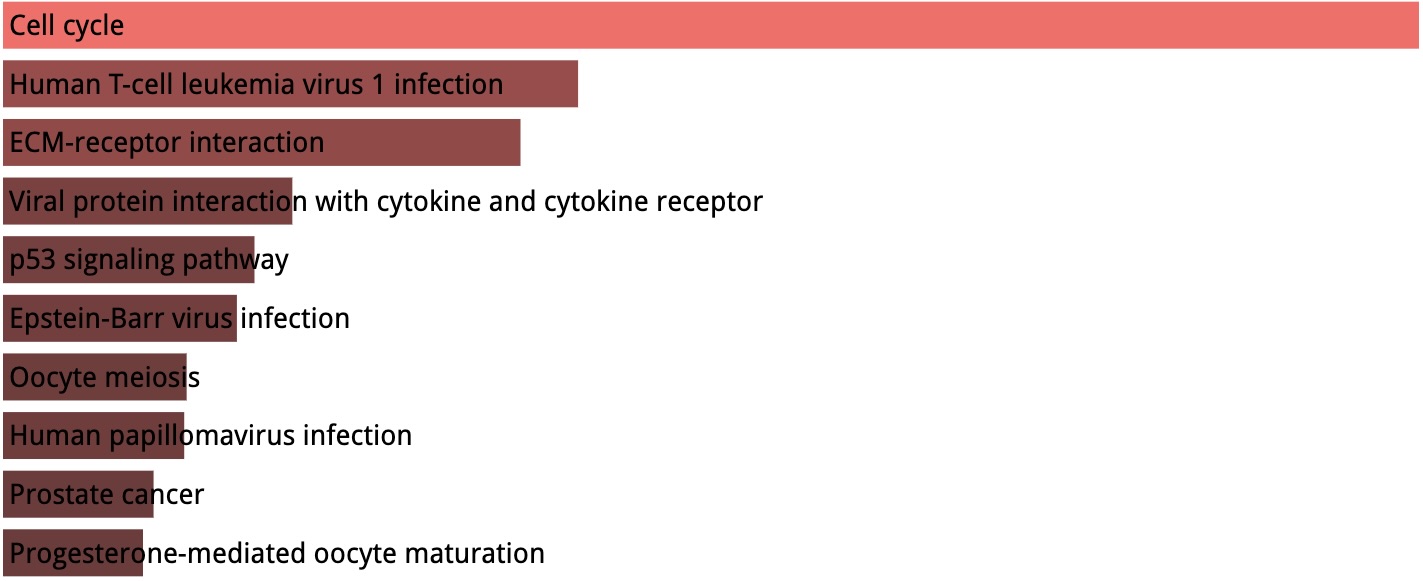 |

**Supplementary Figure 1.** Gene ontology (GO) functional and Kyoto Encyclopedia of Genes and Genomes (KEGG) pathway enrichment analysis for the selected genes (performed via enrichr). (a) GO Biological Process 2021; (b) GO Molecular Function 2021; (c) GO Cellular Component 2021; (d) KEGG 2021.

**Supplementary Tables**

**Supplementary Table 1.** The common DEGs were screened according to adjusted P- values < 0.05 and |logFC| ≥ 1.

| USP21, IDUA, PRUNE1, ZNF580, POGK, ZNF512, PHYHIPL, CKAP4, MPZL1, RFX5, ARHGEF2, TEAD2, SLC1A4, NETO2, AIF1L, RAB11FIP4, ZEB1-AS1, PARD3B, PLK4, HOXB7, BARD1, ANGPT1, TPX2, DEPDC1, LAYN, HEY1, MAB21L2, STX6, PLP2, OSBPL3, KCNJ5, BCL11A, ERP27, ABHD17C, CCND2, FAM171B, FBXO32, DOK5, TLR7, CCNA2, STK39, OXCT1, ZNF738, MBOAT1, AURKA, BCL2L14, ZDHHC13, TYMS, CCNE2, ZNF827, FRMD3, NEDD4L, MCAM, NPAS2, SSPN, SIRPG, MTMR11, LOC374443, MFSD6, STAMBPL1, FBLIM1, PTTG1, SOX9-AS1, TOX, ENPP2, LEF1, CXCL9, DTL, SORT1, STC1, JAG1, CENPE, HLA-DOB, E2F3, RHNO1, NEK2, ANKRD22, SLC7A1, TRAF5, SERPINE2, TCF19, CXCL10, THSD7A, HELLS, LTBP2, INTS6L, PLXNC1, PAQR8, NUSAP1, HOPX, MID1IP1, SNAP25, DKK3 , ENAH, GPNMB, RRM2, RASSF3, MICB, HS3ST2, SOX4, PAQR5, SGO2, CD200, NAV3, XCL1, CDK1, COL5A1, LGR5, GOLM1, FUT4, LAMP3, EFEMP1, MAD2L1, RCAN3, E2F7, PRTFDC1, CDKN2C, RAD51AP1, PDGFA, ZWINT, NCAPG, GLIS2, BHLHE41, SPINT1, MCM10, SOX9, CD3D, PRR11, PFKP, ADAMDEC1, EHF, CDH11, SLFN13, TOP2A, PRKAA2, B3GNT5, APOBEC3B, KDELR3, BUB1B, UHRF1, DTNA, KIF20A, FZD6, EDIL3, HMMR, MGP, GINS2, SMOC2, CCNB2, CDKN3, HKDC1, FAM169A, SLC38A1, ELOVL7, KLHL29, MCM6, DLGAP5, TTK, C12orf75, LRRC1, ANKRD29, CENPU, C15orf48, ANTXR1, DCDC2, CKS2, SLAMF8, COL1A2, RARRES1, PRC1, CXCL11, CDC7, BCAT1, SLC12A2, CCL20, CD24, PBK, ANLN, PDZK1IP1, GINS1, FRAS1, SPP1, VCAN, NPNT, COL15A1, FOXQ1, E2F8, CENPK, FABP4, ASPM, BICC1, CCL18, EPCAM, LY75, FAM3B, COL1A1, THBS2, FNDC1 |
| --- |

**Supplementary Table 2.** Network Specifications of important genes compared with Hub genes

| **Network** | **Network diameter** | **Network density** | **Clustering coefficient** | **Characteristic path on length** |
| --- | --- | --- | --- | --- |
| 49 nodes | 8 | 0.154 | 0.457 | 2.823 |
| 134 nodes | 10 | 0.119 | 0.502 | 3.116 |

**Supplementary Table 3.** 49 Hub genes from the PPI network of differential expressed genes (number of nodes: 49, clustering coefficient: 0.457, network centralization: 0.209)

| MCAM,SOX4, CD3D, CCND2, E2F7, BARD1, ZWINT, CCNE2, ENAH, NPNT, CCNB2, EPCAM, TYMS, FBXO32, NEDD4L, CCNA2, NEK2, BHLHE41, CDK1, SSPN, TOP2A, HMMR, ASPM, COL1A1, COL1A2, LEF1, LGR5, SLAMF8, CXCL9, DTNA, JAG1, SNAP25, THBS2, MGP, SPP1, STX6, CXCL10, ANKRD22, CDKN2C, CCL20, ANLN, VCAN, AURKA, PFKP, CD24, SOX9, STK39, LY75, USP21 |
| --- |
